# Continuity of health care: measurement and application in two rural counties of Guangxi Province, China

**DOI:** 10.1186/s12913-023-09916-4

**Published:** 2023-08-29

**Authors:** Qianchen Wei, Beibei Yuan, Jin Xu, Ping He, Hanyi Xu, Qingyue Meng

**Affiliations:** 1 Department of Health Policy and Management, School of Public Health, Peking University, Beijing, China; 2 China Center for Health Development Studies, Peking University Health Science Center, Beijing, China

**Keywords:** Continuity of care, Composite Indicator, AHP, Rural County

## Abstract

**Background:**

Continuity of care (COC) is highly regarded in health promotion and health system strengthening. However, there is a lack of multidimensional quantitative assessment of continuity, making it challenging to evaluate and compare. Our objective was to create a novel measurement for COC and apply it in two rural counties in China to assess its validity and feasibility in evaluating health system reform.

**Method:**

This study conducted a scoping literature review on COC, examining existing frameworks and indicators. Following an online expert poll, a composite indicator was developed using the analytical hierarchy process (AHP). The measurement tool was then applied to assess the current state of COC in two rural counties in China. In addition to descriptive analysis, demographic and economic characteristics were analyzed for their association with COC scores using t-tests and multiple linear regression models.

**Results:**

The final COC measurement encompasses three dimensions, six sub-dimensions, and ten individual indicators, which integrated and improved the current frameworks and indicators. Relational continuity, informational continuity, and management continuity were identified as the primary dimensions of COC measurement. The COC score is 0.49 in County A and 0.41 in County B, with information continuity being the highest-scoring dimension. Notably, the disparity in continuity scores is most pronounced among individuals with varying attitudes towards health, demonstrating a positive correlation.

**Conclusion:**

The construction of the composite indicator in this study offers a scientific and effective metric for comprehensively measuring continuity of care. The empirical data analysis conducted in Western China serves as an illustrative application of the indicator, demonstrating its efficiency. The results obtained from this analysis provide a solid foundation and valuable reference for strengthening the health system.

**Supplementary Information:**

The online version contains supplementary material available at 10.1186/s12913-023-09916-4.

## Introduction

Continuity of care (COC) is widely recognized as a core component of primary health care and a crucial feature of people-centered integrated care (PCIC). It has been associated with various benefits, including reduced costs [1], increased satisfaction [2], improved health quality and equity [3]. COC is believed to play a pivotal role in addressing the challenges faced by fragmented health delivery systems, ensuring that healthcare services are effectively provided to individuals, which is particularly important for those with non-communicable diseases [4].

Many countries have recognized the significance of COC in their healthcare system reforms, especially in response to challenges posed by epidemiological transitions and population aging [5, 6]. China, as an upper-middle-income country, also confronts similar challenges [7]. Since China launched a comprehensive health reform plan in 2009, the central government to implement a series of policies aimed at improving COC. These policies primarily focus on two key areas. One aims to enhance the capacity of primary care to instill public confidence in healthcare quality [8]. Such policies include forcing high-risk chronic disease patients, the elderly, and other vulnerable populations to contract with the family doctor team [9].

Another area of focus in these policies is the enhancement of coordination among different healthcare providers. This includes the establishment of medical alliances, integrating medical institutions at various levels within a specific region, promoting integrated functions, distinct levels, and resource sharing [10]. These policies have, to some extent, integrated the health system and improved COC. However, due to distorted provider incentives and a weak governance structure, China’s health system still remains hospital-centric, fragmented, and inefficient [11]. In 2016, the report “Deepening Health Reform in China: Building High-Quality and Value-Based Service Delivery” was jointly published by the World Health Organization, World Bank, and the central government (Ministry of Finance, National Health and Family Planning Commission, and Ministry of Human Resources and Social Security). The report proposed the reform of China’s delivery system based on a People-Centered Integrated Care model. Once again, COC was emphasized as a central goal of health reform and the focus of evaluating policy impact [12]. Consequently, a comprehensive and objective measurement of COC has become a crucial issue that needs to be addressed.

Measurement of COC is fundamental for analyzing and formulating policies aimed at improving it. An appropriate measurement tool not only guides the improvement of COC by identifying weak dimensions but also serves as a tracer to monitor and evaluate the progress of reform, providing valuable insights for timely policy adjustments. While researchers have developed various measures of COC, including single indicators like the Usual Provider of Care (UPC) [13] and certain scales [14], these tools have their limitations. Firstly, since the early 21st century, continuity has been widely recognized as a multidimensional concept, and researchers have proposed different theoretical frameworks. However, there has been a lack of quantitative research, with most empirical studies relying on single indicators that do not provide a comprehensive assessment of continuity [13]. Secondly, as society evolves, the health system philosophy is shifting from a “disease-centered” approach to a “people-centered” approach, focusing more on health needs and social determinants. Previous COC measurements typically collected data from the supply side, mainly focusing on medical services, which did not align with the goals of PCIC [15]. Thirdly, past studies have primarily focused on the continuity of primary health care. However, in the context of China, hospitals and primary care centers have overlapping scopes of practice and competition for patients [16]. The lack of coordination between different levels of institutions is an important factor contributing to low continuity in China. Therefore, it is crucial to measure the continuity of the entire health system, taking into account all relevant healthcare providers [17].

The county is the intermediate link from the micro to the macro level of Chinese society, which is the smallest unit for policy formulation and implementation [18]. Attributed to health reform efforts, every county in China has a well-established primary health system and a county-based medical alliance. When calculating the COC score at the county level, the final score encompasses both the continuity of primary health care and medical alliances. In the context of an aging population and the increasing prevalence of chronic diseases, the Chinese government is actively promoting people-centered integrated care to improve health outcomes, reduce inequalities, and save healthcare costs [12]. This need is particularly pronounced in Western China, where economic is less-developed, and rural areas constitute the majority. Consequently, we summarized the characteristics of previous COC frameworks and developed a new measurement tool at the county level, spanning the entire lifespan of the population. Furthermore, we selected two counties in Guangxi, which is a pilot region for people-centered integrated care, as the sample for our empirical study. The objective of this section is not only to assess the feasibility of the new measurement tool for evaluating health system reform but also to provide a model for other researchers to apply this indicator in their studies.

## Method

A COC measurement was developed followed by a empirical study with the measurement method in two counties of Guangxi Province. There are three main steps included in the process of developing the COC measurement in this study.

First of all, we conducted a scoping literature review to explore investigate the definition of COC and compare various frameworks and indicators regarding COC. Electronic database of PubMed, Scopus, Proquest, Google Scholar were used to search English researches in March of 2022. Additionally, we searched Chinese researches, including CNKI, Wanfang and VIP to access relevant Chinese-language research. The following key terms were used: *continuity*, *continuity of care*, *continuity of patient care*, *continuing care*, and *continuum*. The above terms were searched in combination with: *health care*, *defin$*, *dimension$*, *domain$*, *indicator$*, *measur$*, *coordinat$*, and *integrat$*. We thoroughly examined the literature to determine its relevance to the definition or measurement of COC. Based on the findings, this study identified gaps in previous research and developed a new definition and measurement of COC.

Next, the draft of the COC measurement was reviewed by four experts who possess extensive theoretical knowledge and practical experience in the Feld of COC. Four advisory meetings were held to verify the completeness and validity of the indicator’s content. Subsequently, an online expert poll was conducted to further refine the final version of the COC measurement and gather expert opinions on the importance of individual indicators. The questionnaire was emailed to 12 experts in China specializing in health policy and system research between September and November 2021. Analytical hierarchy process (AHP) was chosen as the method to weight and aggregate the composite COC indicator. AHP determines weights based on the relative importance of indicators at each level. Compared to other common weighting methods such as entropy and Delphi, the AHP method assigns weights that better reflect the significance of different indicators and minimizes the influence of subjective factors.

Lastly, the final COC measurement was implemented to assess the level of continuity in two rural counties in Guangxi Province, China. In each county, the division made by local officials into low, middle, and high socioeconomic status (SES) levels was utilized. Random selection was employed to choose one township from each SES stratum. Subsequently, five villages were randomly selected from each chosen township. A total of 600 households were sampled using random sampling methods. The survey encompassed demographic characteristics, health status, and health service utilization. Furthermore, an investigation was conducted on the health centers in the selected townships to gain insights into health service delivery. The cross-sectional survey was carried out in July 2021. This study employed descriptive analysis to depict the overall COC score and different dimensions in the two counties, enabling a comparison between them. COC scores were compared across various demographic and economic characteristics using t-tests. Multiple linear regression models were used to examine the association between COC scores and demographic and economic characteristics controlling for confounders.

## Results

### Review of previous frameworks on COC

**Fig. 1:**
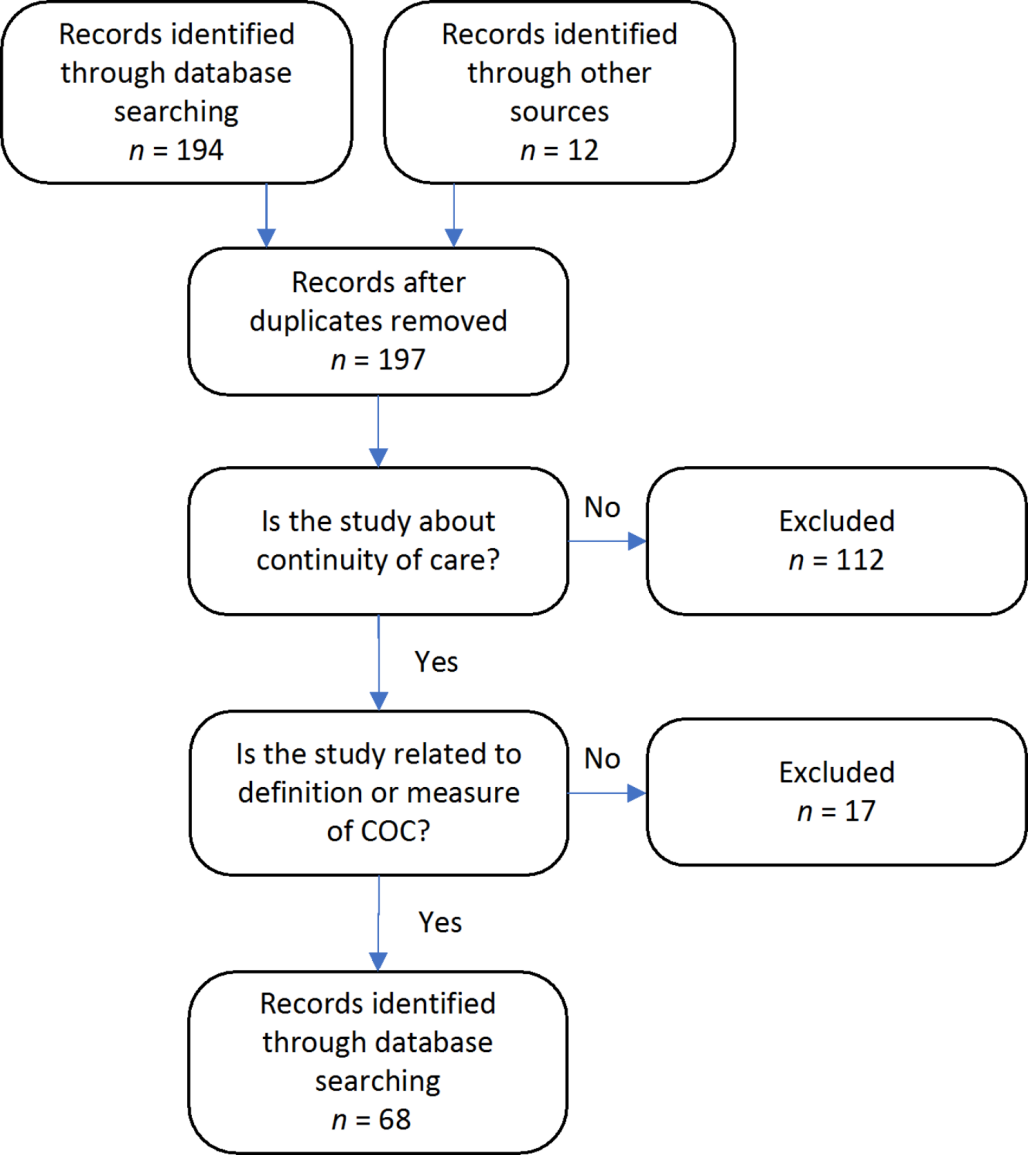
PRISMA flowchart of study selection process

The original search returned 206 articles, 68 of which were procured for review after deduplication and relevance screening. The flow of articles through identification to final inclusion is represented in Fig. 1.

In reviewing the literature on COC, it became apprarent that COC is a complex and comprehensive concept. It was first proposed in the 1960s in the Folsom Report, which defined COC as “maximizing the services received by health service users to be provided by the same health service provider” [19]. With the highly specialized development of healthcare, individuals now need to engage with multiple types of providers throughout their healthcare journey, especially for patients with chronic or comorbid conditions. Consequently, the definition of health service continuity has also evolved. As defined by Haggerty, COC is “the consistency and coherence of a series of discrete healthcare events with the needs of patients” [20]. This definition emphasizes two core elements: the focus on health service users and the provision of long-term and multiple services to meet their needs [21].

We found 26 frameworks proposed by international and Chinese researchers, each comprising one or more dimensions (Appendix 1). The UPC and Bice-Boxerman COC are commonly used in empirical studies due to their ease of measurement. However, these frameworks only capture a single aspect of COC and fail to provide a comprehensive picture [13]. Among the multi-dimensional frameworks, Haggerty’s framework of COC, which includes relational, informational, and management continuity, is widely cited. This framework offers multiple measurable dimensions that reflect the complexity of COC. However, all the indicators mentioned in this framework are objective in nature [22]. In light of the evolving concept of health services from a disease-centered approach to a people-centered approach, Freeman introduced the concept of “experienced continuity” as the ultimate goal of COC [23]. The World Health Organization (WHO) developed a framework for COC consisting of four domains: interpersonal, longitudinal, management, and informational continuity, which incorporates both subjective and objective indicators. However, the WHO framework primarily focuses on medical care and overlooks the continuity of prevention services, which is particularly important in the context of the increasing prevalence of chronic diseases [21]. While there are measurement tools that encompass more dimensions, such as the Nijmegen scale [14] the associated questionnaires can be overly complex and difficult to comprehend, particularly for individuals with limited education.

In conclusion, COC is a multi-dimensional concept that signifies residents’ ability to access appropriate, seamless, and satisfactory health services, encompassing both medical care and other healthcare needs, based on their individual health requirements and personal circumstances, provided by a consistent team of healthcare professionals. To facilitate measurement, it is advisable to minimize the number of dimensions and avoid redundancy. Considering these findings, relational continuity, informational continuity, and management continuity were chosen as the primary elements in the measurement of COC. Interpersonal continuity and longitudinal continuity were included as sub-dimensions within relational continuity, while experience continuity was incorporated within the domain of management continuity [24].

### Development of the COC measurement

#### Selection of indicators

Based on literature review and expert consultation, the final measurement of COC consists 3 dimensions, 6 variables and 10 individual indicators, as shown in Table 1:

**Table 1:** Stucture of the COC measurement

Dimension	Variable	Individual Indicator	Description
Relational Continuity	Fixed Relationship	Family doctor contracts	Whether the household contract with a family doctor in form
		Proportion of visits to the same doctor	Sum of squared ratio of a person visits the certain doctor to the total visits in 12 months
	Quality of relationship	Rapid response	Whether residents can contact the family doctor quickly in need
		Satisfaction	The score of satisfaction in contact with doctors
Informational Continuity	Accumulated Knowledge	Access to health information	Whether the health center has established electronic health record
		Access to personal information	Whether the professional obtain the psychosocial information
	Information Transfer	Information transfer	Whether information can be shared between different healthcare providers
Management Continuity	Consistency of care	Access to health resources	Whether people obtain the same high-quality services from different professionals
		Consistency of treatment	Whether the cross-boundary health service is coherent
	Flexibility	Patient participation	Whether the patient participated in the development of treatment plans

##### Relational continuity

This dimension focuses on establishing a consistent and ongoing relationship between residents and one or more healthcare providers who are familiar with each other. This dimension is disassembled into two aspects: (1) Fixed relationship reflects the objective continuity, captured using two indicators that *family doctor contracts* in form and *proportion of visits to the same doctor* in actual visits; (2) Quality of relationship is seclected as the subjective variable, which assessed by *responsibility capability* of family doctor and *satisfaction* evaluated by individuals on experience in interacting with doctors.

##### Informational continuity

This dimension focuses on developing appropriate health management plans based on health information and personal information of the residents, regardless of time and location. This dimension consists two components: (1)*accumulated knowledge* is derived by measuring *access to health information and personal information*, which reflects previous medical records and psychosocial characteristics; (2)*information transfer* is considered the access of individuals’ previous information in the context of cross-boundary collaboration.

##### Management continuity

This dimension emphasizes establishing a well-organized cooperation mechanism between different health service institutions to meet the envolving health needs of residents. It comprises two items: (1)The *consistency of care* refers to consistency of care and a smooth transition process, captured using *access to health resources* and *consistency of treatment*. (2)The flexibility of care highlights adapting care to changes in an individual’s needs and circumstances, which is measured by *patient participation*.

#### Weighting and aggregation

##### Weighting

The weights of the individual indicators were determined using the AHP method. A total of 12 experts participated in scoring the indicators, resulting in a response rate of 67.7% (The questionnaire presented in Appendix 2). The relative weights of the individual indicators were calculated using an eigenvector, as shown in Table 2. To ensure the accuracy of the judgments, the inconsistency ratios of all comparison matrices were found to be less than 0.1, meeting the consistency requirements.

**Table 2:** Weights of the indicators for COC

Dimension	Weight(%)	Variable	Weight(%)	Individual Indicator	Weight(%)
Relational Continuity	60.43	Fixed Relationship	46.87	Family doctor contracts	33.33
				Proportion of visits to the same doctor	13.54
		Quality of relationship	13.56	Rapid response	9.46
				Satisfaction	4.10
Information Continuity	25.14	Accumulated Knowledge	15.69	Access to health information	11.35
				Access to personal information	4.33
		Information Transfer	9.41	Information transfer	9.41
Management Continuity	14.43	Consistency of care	11.82	Access to health resources	8.31
				Consistency of treatment	3.51
		Flexibility	2.58	Patient participation	2.58

Since improvements in each dimension have a spillover effect, different dimensions and indicators within the same dimensions compensate for each other. To synthesize the composite indicator for COC, linear aggregation is applied, which is a common compensatory aggregation approach that allows for trade-offs between different indicators [25]. For an individual $k$, the composite indicator for COC can be calculated using the following formula:

$${COC}_{k}=\sum _{i=1}^{n}{W}_{i}{\lambda }_{i}\,\,k=1,\,..., n$$

where ${\lambda }_{i}$ is the standardized value of individual indicator $i$ by min-max algorithm, ${W}_{i}$ is the weight of indicator $i, n$ is the number of individual indicators.

##### Reliability and validation

The composited indicator demonstrates strong reliability, as indicated by a Cronbach’s α index of the indicator is 0.85 (p < 0.05). To validate the measurement, a principal component analysis (PCA) was conducted. Three principal components with eigenvalues greater than 1 are extracted, and the cumulative contribution rate of variance reached 82.21%, indicating the validity of the measurement (Appendix 3).

##### Measures of COC score

In accordance with the needs of policy application, the measurements of COC are divided into two categories: (1) overall score at the population level: calculated by averaging the COC scores, allowing for horizontally comparisons between different regions; (2) distribution of COC scores: representing the density of COC scores within the range of 0–1, enabling the identification of health system equity and population disparities.

### Level of COC in two rural counties based on the COC measurement

#### Data description

Multiple visits across time are a prerequisite for continuity measurement. For the calculation of indicators, samples that received health services two or more times within 12-month period were included in this study. There were no patients with missing data that needed to be excluded. The final data available for analysis in this study consist of 251 individuals and 12 health centers. The characteristic of selected samples were shown in Table 3. Among them, 48.6% were from County A and 51.4% from County B. The proportion of male is 51% and the average age is 56.93 years. The educational level of the population is moderate, with only 7.2% having a high school degree or above. The majority of samples had social health insurance. The income distribution of 251 samples included in this study is consistent with population, with an average income of 11814.6 RMB per year.

**Table 3:** Summary statistics of the samples

Variables	Number	Proportion(%)
**County**		
County A	122	48.6
County B	129	51.4
**Age**		
<65	167	66.5
65~	84	33.5
**Gender**		
Male	128	51.0
Female	123	49.0
**Education**		
Primary school and below	131	52.2
Junior school and above	120	47.8
**Income**		
Low income	40	20.7
High income	211	19.5
**Health Insurance program**		
Social health insurance	246	98.0
Self-pay	5	2.0
**Attitude towards health**		
Not important	40	15.9
Important	211	84.1

Due to the unavailability of certain variables in Guangxi, we opted for alternative indicators. Considering that doctors play a primary role in delivering health services and the policy encourages specialists from specialized hospitals to establish outpatient clinics at primary care facilities, we selected “whether township health centers have set up specialist outpatient clinics” to demonstrate the access to health resources [26]. Additionally, the population in the two counties generally lacks education, particularly in terms of medical knowledge, makes it challenging for them to actively participate in decision-making. Therefore, we used the adequacy of communication in health care as a proxy variable for patient participation [27]. Higher levels of thorough communication indicate greater patient participation.

#### The overall score of COC

Table 4 presents the COC scores in the two counties. Assessing the level of continuity of care and conducting a disaggregated analysis allows for meaningful comparisons and serves as an essential tool for evaluating the progress of PCIC. Understanding the differences between the counties is of practical significance for identifying policy priorities in the future. The disparities between the two counties were measured using *t*-tests, and the results are discussed below.

The average score of COC in County A is 0.49, while County B is 0.41, indicating that both counties are at a medium level of continuity. Further analysis reveals that the weak points of COC in both counties are relatively similar. Among the dimension indicators, the largest gap between the two counties is observed in relational continuity, with County A scoring 0.12 higher than County B. Among the dimension indicators, This is followed by management continuity, with a gap of 0.08. On the other hand, the difference in informational continuity is minimal and statistically insignificant. Regarding the sub-indicators, except for accumulated knowledge and flexibility, County A consistently receives significantly higher scores than County B. The largest discrepancy is observed in the fixed relationship indicator, where County A scores 0.13 higher than County B.

**Table 4:** Results of COC Score in the Two Counties

	Score of County A, Mean(SD)	Score of County B, Mean(SD)
**COC score**	0.49(0.22)	0.41(0.18)
Relational Continuity	0.49(0.34)	0.37(0.30)
*Fixed Relationship*	0.50(0.35)	0.38(0.31)
*Quality of relationship*	0.46(0.36)	0.35(0.29)
Information Continuity	0.64(0.19)	0.62(0.19)
*Accumulated Knowledge*	1.00(0.00)	1.00(0.00)
*Information Transfer*	0.50(0.50)	0.45(0.50)
Management Continuity	0.27(0.28)	0.19(0.23)
*Consistency of care*	0.19(0.32)	0.11(0.26)
*Flexibility*	0.63(0.34)	0.58(0.33)

#### Distribution of COC score

To examine the distribution of COC scores among different groups, we conducted an analysis based on age, gender, education, income, and attitude towards health. The findings are presented in Appendix 5. The results indicate that individuals aged over 65, those with lower income, individuals who prioritize health, and residents of County A obtained higher COC scores. Additionally, Table 5 presents the regression results for COC scores. The analysis reveals significant differences in COC scores based on individuals’ attitudes towards health. Specifically, individuals who place greater emphasis on health demonstrate a higher level of continuity (p < 0.001). Consistent with the earlier findings, residents of County A also exhibit significantly higher COC scores compared to those in County B. Furthermore, when controlling for confounding variables, the effects of age and income on COC scores no longer remain significant. This suggests that age and income do not independently influence continuity and their impact can be attributed to other factors.

**Table 5:** Regression results on characteristics in COC score

Characteristics	Coef.	95% CI
Gender (ref: male)		
female	0.02	[-0.03, 0.07]
Age (ref: <65)		
65~	0.04	[-0.02, 0.09]
Education (ref: primary school and below)		
junior school and above	-0.02	[-0.07, 0.04]
Income (ref: low income)		
high income	-0.01	[-0.06, 0.04]
Attitude towards health (ref. not important)		
important	0.10^***^	[0.03, 0.17]
County (ref: County A)		
County B	-0.08^***^	[-0.14, -0.03]

*** p < 0.01, ** p < 0.05, * p < 0.1

## Discussion

In this study, we have developed a comprehensive and quantitative measure of continuity based on a multidimensional framework. By consulting with experts in the field of health policy, we have constructed a composite indicator that is both robust and easily measurable, using AHP method. The validity of the measurement has been confirmed through reliability analysis and principal component analysis. The new measurement captures the complexity of continuity by incorporating multiple dimensions and indicators that reflect various aspects of the broader health system. The weighting of each indicator is also meaningful, as it provides guidance for prioritizing areas in health reform and policy development. Furthermore, we have demonstrated the practical application of the new measure in this study. The multidimensional measurement enables us to identify weak points and areas requiring improvement, thus informing policy priorities. Additionally, by analyzing the distribution of COC scores among the population, we can identify vulnerable groups and tailor policies to enhance health equity.

In the subsequent discussion, we will focus on the selection of indicators, the rationale behind the assigned weights, and the empirical results to further analyze the validity and applicability of the new measurement. Moreover, we will explore how the COC scores can be effectively linked to health policies to drive improvements in continuity of care.

Firstly, the dimension of relational continuity carries the highest weight in the measurement, with a particular emphasis on the indicator called “family doctor contracts,“ which is considered the most crucial among all indicators. In China, family doctors primarily consist of rural doctors who have been transformed into this role. According to policy requirements, family doctors are obligated to contract with all chronic patients and individuals aged 65 and above within their designated area. Additionally, a specified number of follow-up visits within a year is mandated. The results of this indicator also highlight the policy differences between the two counties [28]. County A allows a greater number of ordinary residents to contract with family doctors, which is the primary reason for its significantly higher score in the fixed relationship indicator compared to County B. However, it is worth noting that the overall scores for this indicator in both counties are not satisfactory, particularly regarding the quality of the relationship. These findings align with other studies that have reported a low utilization rate of family doctor contracting services in rural areas of Guangxi, which stands at a mere 6.7% [29]. This low utilization may be attributed to the substantial overlap between the content of family doctor contracting services and basic public health services under current policies. Consequently, many people are unaware that they have actually contracted with a family doctor and thus rarely seek assistance from them.

Secondly, information continuity achieved the highest score among the three dimensions, and the difference between the two sample counties is relatively small. This score can be attributed to the comprehensive implementation of electronic medical records at the township level, which represents a significant accomplishment in the rural areas of western China [30]. It demonstrates the dedication and efforts of the health departments in promoting COC. By 2019, approximately 82.82% of primary healthcare facilities nationwide had implemented information systems [31]. The utilization of information technology serves as a foundation for personalized health services and plays a crucial role in improving health literacy. However, the score for information transfer indicates that there is poor interoperability among health information systems. Fragmentation is evident not only within electronic medical record systems across different hospitals but also in the connection between clinical care and basic health services. This highlights the need for improved integration and coordination of health information systems to enhance information transfer and facilitate seamless care delivery [32].

Thirdly, management continuity is identified as the weakest dimension of COC in both counties, particularly in terms of consistency of care. The establishment of medical alliances in the two rural counties has made certain contributions to integrated care, and the discrepancy in scores clearly indicates the better performance of County A in terms of coordination. However, similar to the policy pilot experiences in other areas of China, there is a lack of consensus among the stakeholders within the medical alliances [33]. During the long-term process of policy design and implementation, the coordination processes within county healthcare alliances have been unclear and inadequately supported by funding. Hospitals and primary care centers have been engaged in patient competition and lacked incentives to collaborate, thereby hindering the continuity of management [16]. Additionally, due to insufficient promotion efforts, most residents still harbor a distrust of the quality of primary healthcare services and continue to flock to large hospitals, overlooking the government’s efforts to improve healthcare accessibility. These findings align with the results of another empirical study conducted in western China, which confirmed that 44% of respondents were unaware of the policies related to the tiered healthcare delivery system [34]. The lack of awareness and understanding among residents further hampers the successful implementation of management continuity initiatives.

The distribution of COC scores revealed significant variations in continuity among individuals with different attitudes towards health. Those who prioritize their health tend to be more proactive in managing their health conditions and are more likely to establish a stable relationship with healthcare providers to ensure ongoing guidance and support. They exhibit a greater interest in favorable health policies that enhance relational continuity and management continuity. These findings emphasize the importance of government initiatives aimed at health promotion and improving the overall health literacy of the population. By enhancing health literacy, individuals can make informed decisions about their healthcare, actively engage in preventive measures, and effectively utilize available healthcare resources to enhance continuity of care.

Our empirical findings have significant policy implications. Firstly, the dimension of relational continuity emerges as the most crucial aspect of COC [35]. Therefore, it is necessary to reevaluate the family doctor contracting policy and emphasize its distinctive features [36]. It is necessary to improve the capacity of primary health care providers and strengthen policy publicity that guide more people, not only those with chronic diseases and the elderly, to contract with family doctors. Secondly, while both counties exhibit high scores in informational continuity, the lack of integrated data hinders the provision of seamless services, particularly in underdeveloped regions like Guangxi. To address this issue, the government should recognize the potential of information technology in compensating for transportation challenges and allocate additional funding for telemedicine initiatives to enhance access to care [37]. Thirdly, efforts have been made in Guangxi to improve management continuity through the establishment of medical alliances. However, compared to cities like Sanming and Luohu [38], the progress is still in its early stages [39]. It is crucial to learn from successful integration experiences and promote inter-institutional collaboration at different levels to enhance the continuity of health services in Guangxi [40]. Lastly, raising public awareness is paramount in Guangxi. This includes fostering knowledge about health and related policies among the population, enabling them to make optimal use of available resources and improve continuity of care. These policy implications emphasize the importance of refining existing policies, addressing information gaps, promoting collaborative healthcare models, and enhancing public awareness to advance continuity of care in Guangxi.

One significant limitation of our study is the small sample size. Continuity of care is demonstrated by observing multiple instances of receiving health services over time [20]. Although we only had a sample of 251 individuals included in our empirical study, we were still able to identify significant differences in COC scores between counties and among individuals with different attitudes towards health. With a larger sample size, it would have been easier to identify vulnerable populations and provide more precise insights. Furthermore, the data used in our study were obtained from a cross-sectional survey, which limits our analysis to descriptive and comparative approaches. It does not allow us to examine temporal trends or make causal inferences. To evaluate the effects of healthcare reforms, future studies should incorporate longitudinal data and include comparisons across different time points. Addressing these limitations and expanding the sample size in future studies would enhance the robustness and generalizability of the findings, allowing for a more comprehensive understanding of continuity of care and its implications for health system improvements.

## Conclusion

This study contributes to bridging the gap between the theoretical framework and quantitative measurement of continuity of care by introducing a composite indicator that encompasses the various dimensions of COC. The measurement results not only provide insights into the effectiveness of health policies concerning continuity of care but also offer a valuable foundation for the optimization of these policies by identifying areas of strength and weakness in COC.

### Electronic supplementary material

Below is the link to the electronic supplementary material.

Supplementary Material 1

Supplementary Material 2

Supplementary Material 3

Supplementary Material 4

Supplementary Material 5

## Data Availability

The datasets generated during the current study are not publicly available because some of the data may related to personal privacy, which are available from the corresponding author on reasonable request. And all data analysed during this study are included in this published article.
