# Supplementary material for: Continuity of health care: measurement and application in two rural counties of Guangxi Province, China
Source: BMC Health Serv Res. 2023 Aug 29;23:917. doi: 10.1186/s12913-023-09916-4 (PMC10464216; doi:10.1186/s12913-023-09916-4)
Supplement: Supplementary file 1 — Supplementary Material 1 [file 12913_2023_9916_MOESM1_ESM.docx]

**Appendix 1:** The COC frameworks with 1-7 dimensions

| Number of  dimensions | Number of  combinations | Dimensions |
| --- | --- | --- |
| 1 dimension | 5 combinations | duration; density (e.g., usual provider care, UPC); dispersion (e.g., Bice-Boxerman continuity of care index, COC); sequence; subjective estimates. |
| 2 dimensions | 3 combinations | longitudinal continuity and whether having a personal doctor; management continuity and informational continuity; horizontal continuity and vertical continuity |
| 3 dimensions | 9 combinations | relational continuity, informational continuity and management continuity; interpersonal continuity, management continuity and informational continuity; longitudinal continuity, patient–professional relationships, and coordinated care; system access, interpersonal aspects, and care team function; access to services, breaks in service delivery and contact with particular professionals; Usual Provider Continuity, length of time with regular physician and importance of seeing regular physician; personal continuity, personal continuity, cross-boundary continuity; cross-sectional continuity, longitudinal continuity and relational continuity; longitudinal care, consultation experiences and patient-doctor depth of relationship. |
| 4 dimensions | 4 combinations | interpersonal continuity, longitudinal continuity, informational continuity and management continuity; longitudinal continuity, relational continuity, flexible continuity and cross-boundary continuity; relational continuity, institutional continuity, disciplines continuity and informational continuity; relational continuity, management continuity, information continuity and flexible continuity. |
| 5 dimensions | 2 combinations | access to services, interactions with physician, interactions with other health care providers, personal self-responsibility, and communication; relationship, timeliness, mutuality, choice and knowledge. |
| 6 dimensions | 1 combination | pinch hitting, trouble shooting, smoothing transitions, creating flexibility, speeding the system up, and contextualizing |
| 7 dimensions | 2 combinations | longitudinal, individual, comprehensiveness, flexibility, relationship, accessibility and communication; chronology, geography, specialty, relationship, information, accessibility and mobility; experience and relationships, regularity, meeting needs, consolidation, managed transitions, care Coordination, supported living. |
