# Supplementary material for: Continuity of health care: measurement and application in two rural counties of Guangxi Province, China
Source: BMC Health Serv Res. 2023 Aug 29;23:917. doi: 10.1186/s12913-023-09916-4 (PMC10464216; doi:10.1186/s12913-023-09916-4)
Supplement: Supplementary file 2 — Supplementary Material 2 [file 12913_2023_9916_MOESM2_ESM.docx]

**Appendix 2: AHP Questionnaire**

| Circle one number per row below using the scale:  1 = Equal  3 = Moderate  5 = Strong  7 = Very strong  9 = Extremely strong | | | |
| --- | --- | --- | --- |
| 1 | Relational continuity | 9 8 7 6 5 4 3 2 1 2 3 4 5 6 7 8 9 | Informational Continuity |
| 2 | Relational continuity | 9 8 7 6 5 4 3 2 1 2 3 4 5 6 7 8 9 | Management Continuity |
| 3 | Informational Continuity | 9 8 7 6 5 4 3 2 1 2 3 4 5 6 7 8 9 | Management Continuity |
| **Relational continuity** | | | |
| 4 | Fixed Relationship | 9 8 7 6 5 4 3 2 1 2 3 4 5 6 7 8 9 | Quality of relationship |
| 5 | Family doctor contracts | 9 8 7 6 5 4 3 2 1 2 3 4 5 6 7 8 9 | Proportion of visits to the same doctor |
| 6 | Rapid response | 9 8 7 6 5 4 3 2 1 2 3 4 5 6 7 8 9 | Satisfaction |
| **Informational continuity** | | | |
| 7 | Accumulated Knowledge | 9 8 7 6 5 4 3 2 1 2 3 4 5 6 7 8 9 | Information Transfer |
| 8 | Access to health information | 9 8 7 6 5 4 3 2 1 2 3 4 5 6 7 8 9 | Access to personal information |
| **Management continuity** | | | |
| 9 | Consistency of care | 9 8 7 6 5 4 3 2 1 2 3 4 5 6 7 8 9 | Flexibility |
| 10 | Access to health resources | 9 8 7 6 5 4 3 2 1 2 3 4 5 6 7 8 9 | Consistency of treatment |
