# Supplementary material for: Continuity of health care: measurement and application in two rural counties of Guangxi Province, China
Source: BMC Health Serv Res. 2023 Aug 29;23:917. doi: 10.1186/s12913-023-09916-4 (PMC10464216; doi:10.1186/s12913-023-09916-4)
Supplement: Supplementary file 3 — Supplementary Material 3 [file 12913_2023_9916_MOESM3_ESM.docx]

**Appendix 3: The result of Principle Component Analysis**

| Dimension | Variable | Component loadings | | | cumulative variance contribution rate |
| --- | --- | --- | --- | --- | --- |
|  |  | Component 1 | Component 2 | Component 3 |  |
| Relational continuity | Fixed Relationship | 0.940 |  |  | 39.33% |
|  | Quality of relationship | 0.951 |  |  |  |
| Informational continuity | Accumulated Knowledge |  | /^*^ |  | 59.92% |
|  | Information Transfer |  | 0.838 |  |  |
| Management continuity | Consistency of care |  |  | 0.625 | 82.21% |
|  | Flexibility |  |  | 0.806 |  |

^*^The scores for the variable “accumulated Knowledge” are both 1
