# Supplementary material for: Continuity of health care: measurement and application in two rural counties of Guangxi Province, China
Source: BMC Health Serv Res. 2023 Aug 29;23:917. doi: 10.1186/s12913-023-09916-4 (PMC10464216; doi:10.1186/s12913-023-09916-4)
Supplement: Supplementary file 4 — Supplementary Material 4 [file 12913_2023_9916_MOESM4_ESM.docx]

Appendix 4: Sensitivity Analysis

To evaluate how much the uncertainties of input factors affect the total variance of our composite indicator, we perform a sensitivity analysis based on Sobol’ sensitivity measures with a computationally improved form developed by Saltelli^1^. The 12 uncertain input factors with their associated PDF are described in Table s1, according to which a total number of 10000 Monte Carlo simulations are performed. For each simulation, $X_{1}-X_{12}$ are sampled from their distributions and are used to calculate a COC score as an output. Then we can obtain first order effects $S_{i}$ and total effects $S_{Ti}$ after 10000 simulations. The results are presented in Table s2. It can be seen that family doctor contracts, access to health resources are important variables, as well as normalization method. When taken singly, all the input factors can explain 81% of the output variance. The weighting scheme has little effect on the output singly but has a relatively strong interaction with other factors as indicated by its relatively high $S_{Ti}-S_{i}$ value.

**Table s1** The 12 uncertain input factors

| Input factor | Definition | PDF | Range |
| --- | --- | --- | --- |
| $X_{1}$ | Family doctor contracts | Discrete, uniform | [0;1] |
| $X_{2}$ | Herfindahl–Hirschman Index | Continuous, uniform | [0,1] |
| $X_{3}$ | Rapid response | Discrete, uniform | [0;1] |
| $X_{4}$ | Satisfaction | Continuous, uniform | [0,1] |
| $X_{5}$ | Access to health information | Discrete, uniform | [0;1] |
| $X_{6}$ | Access to personal information | Discrete, uniform | [0;1] |
| $X_{7}$ | Information transfer | Discrete, uniform | [0;1] |
| $X_{8}$ | Access to health resources | Discrete, uniform | [0;1] |
| $X_{9}$ | Consistency of treatment | Discrete, uniform | [0;1] |
| $X_{10}$ | Patient participation | Continuous, uniform | [0,1] |
| $X_{11}$ | Weighting scheme | Uniform | [0,1], where [0,0.5) $\equiv$ equal weights $(W_{i}=0.1 for all indicators)$ and [0.5,1] $\equiv$ AHP weighting (Table 1). |
| $X_{12}$ | Normalization method | Uniform | [0,1], where [0,0.5) $\equiv$ standardized values and [0.5,1] $\equiv$ rescaled values. |

**Table s2** Sensitivity measures of first-order and total effects for COC scores

| Input factor | First-order effect $(S_{i})$ | Total effect $(S_{\mathrm{Ti}})$ | $S_{\mathrm{Ti}}-S_{i}$ |
| --- | --- | --- | --- |
| $X_{1}$ | 0.22 | 0.33 | 0.11 |
| $X_{2}$ | 0.05 | 0.07 | 0.02 |
| $X_{3}$ | 0.07 | 0.08 | 0.01 |
| $X_{4}$ | 0.03 | 0.05 | 0.02 |
| $X_{5}$ | 0.03 | 0.04 | 0.01 |
| $X_{6}$ | 0.01 | 0.02 | 0.01 |
| $X_{7}$ | 0.06 | 0.07 | 0.01 |
| $X_{8}$ | 0.10 | 0.12 | 0.02 |
| $X_{9}$ | 0.04 | 0.06 | 0.02 |
| $X_{10}$ | 0.03 | 0.03 | 0.00 |
| $X_{11}$ | 0.00 | 0.11 | 0.11 |
| $X_{12}$ | 0.18 | 0.31 | 0.13 |
| Sum | 0.81 | 1.29 | 0.48 |

Reference

1. Saltelli A. Making best use of model evaluations to compute sensitivity indices. *Computer Physics Communications*. 2002;145(2):280-297. doi:10.1016/S0010-4655(02)00280-1
