# Supplementary material for: Continuity of health care: measurement and application in two rural counties of Guangxi Province, China
Source: BMC Health Serv Res. 2023 Aug 29;23:917. doi: 10.1186/s12913-023-09916-4 (PMC10464216; doi:10.1186/s12913-023-09916-4)
Supplement: Supplementary file 5 — Supplementary Material 5 [file 12913_2023_9916_MOESM5_ESM.docx]

**Appendix 5:** COC score with different characteristics

| **Variables** | **COC score, mean(SD)** | **P-value** |
| --- | --- | --- |
| **County** |  |  |
| County A | 0.50(0.22) | 0.00 |
| County B | 0.41(0.18) |  |
| **Age** |  |  |
| <65 | 0.43(0.21) | 0.03 |
| 65~ | 0.49(0.20) |  |
| **Gender** |  |  |
| Male | 0.45(0.20) | 0.67 |
| Female | 0.46(0.21) |  |
| **Education** |  |  |
| Primary school and below | 0.46(0.21) | 0.27 |
| Junior school and above | 0.44(0.21) |  |
| **Income** |  |  |
| Low income | 0.46(0.20) | 0.01 |
| High income | 0.45(0.21) |  |
| **Attitude towards health** |  |  |
| Not important | 0.41(0.18) | 0.01 |
| Important | 0.46(0.22) |  |
